# Pulmonary Fibrosis in a Patient With a Prolactinoma on Dopamine Agonists: Coincidence or Consequence

**DOI:** 10.1210/jcemcr/luaf067

**Published:** 2025-04-15

**Authors:** Ravi Shah, Amanjit Bal, Durairaj Arjunan, Jayaditya Ghosh, Ashley B Grossman, Pinaki Dutta

**Affiliations:** Department of Endocrinology, PGIMER, Chandigarh 160012, India; Department of Histopathology, PGIMER, Chandigarh 160012, India; Department of Endocrinology, PGIMER, Chandigarh 160012, India; Department of Endocrinology, PGIMER, Chandigarh 160012, India; Green Templeton College, University of Oxford, Oxford OX1 2JD, UK; Centre for Endocrinology, Barts and the London School of Medicine, Queen Mary University of London, London E1 4NS, UK; NET Unit, ENETS Centre of Excellence, Royal Free Hospital, London NW3 2QG, UK; Department of Endocrinology, PGIMER, Chandigarh 160012, India

**Keywords:** prolactinoma, ergot-derived dopamine agonists, cabergoline, bromocriptine, interstitial lung disease, pulmonary fibrosis

## Abstract

Prolactinomas are the most common functional pituitary tumor and are typically managed with dopamine agonists such as bromocriptine or cabergoline. Although these agents are generally well tolerated and effective in reducing prolactin levels and often tumor size, they have been implicated in rare but serious fibrotic complications, including interstitial lung disease (ILD). We describe a 65-year-old man with a longstanding prolactinoma who received cumulative high-dose bromocriptine and cabergoline therapy over several decades. Despite initial tumor shrinkage and partial biochemical control of hyperprolactinemia with dopamine agonists, stereotactic radiosurgery, and transsphenoidal surgery, the patient developed progressive exertional dyspnea and cough, accompanied by imaging and histopathological findings consistent with “usual interstitial pneumonia” (UIP). Autoimmune and environmental causes were largely excluded, suggesting a drug-induced etiology.

Following discontinuation of cabergoline, the patient has been on continued surveillance of his prolactin levels and tumor status, with symptomatic treatment of his UIP.

This case underscores the potential for dopamine agonist–associated ILD, even in patients with prolactinomas who generally receive lower weekly doses than those used in Parkinson’s and related diseases. Early recognition of respiratory symptoms, pulmonary function, and radiological investigations are indicated in selected symptomatic cases.

## Introduction

Cabergoline is the preferred first-line dopamine agonist for prolactinoma compared to bromocriptine (BRC), effectively lowering prolactin (PRL) levels and reducing tumor size [1, 2]. While generally well tolerated, dopamine agonists can cause nausea, orthostatic hypotension, impulse control disorders, and, less commonly, fibrotic complications like cardiac valvulopathy and pulmonary fibrosis, especially at high doses [3]. Interstitial lung diseases (ILD) include inflammatory and fibrotic lung disorders, sometimes drug-induced. While agents like amiodarone and bleomycin are known triggers, dopamine agonist-induced pulmonary fibrosis is rare and underrecognized [4, 5].

We report a prolactinoma patient on long-term dopamine agonist therapy who developed ILD with a usual interstitial pneumonia (UIP) pattern, previously noted for impulse control disorder related to treatment.

## Case Presentation

A 65-year-old man presented in 2003 with a history of headaches, diminished vision, loss of libido, and erectile dysfunction since the age of 30 years. At the age of 44 years, he was evaluated and found to have a sellar mass measuring 4.2 × 3.6 × 2.9 cm, with suprasellar and bilateral para-sellar extension. Hormonal evaluation revealed hyperprolactinemia with serum PRL levels of 5200 ng/mL [International System of Units (SI): 110240 mIU/L] (reference range: 4.79-23.3 ng/mL (SI: 101.54-493.96 mIU/L)] and secondary hypogonadism. He was euthyroid and had borderline cortisol reserve. (Table 1)

**Table 1.** Hormonal profiles and imaging findings over time

Parameter	March 3	June 3	November 9	November 17	March 18	May 24	Reference range
PRL	5200 ng/mL, (SI: 110240mIU/L)	1233 ng/mL, (SI: 26139.6 mIU/L)	5200 ng/mL, (SI: 110240 mIU/L)	503 ng/mL, (SI: 10663.6 mIU/L)	218 ng/mL, (SI: 4621.6 mIU/L)	5 ng/mL, (SI: 106 mIU/L)	4.79-23.3 ng/mL, (SI: 101.54-493.96 mIU/L)
LH	1.1 mIU/mL (conventional and SI)			0.1 mIU/L (conventional and SI)			2.4-12.6 mIU/mL (conventional and SI)
FSH	1 mIU/L (conventional and SI)			1.57 mIU/mL (conventional and SI)			3.5-12.5 mIU/mL (conventional and SI)
Testosterone	230 ng/dL (SI: 8 nmol/L)		135.36 ng/dL (SI: 4.7 nmol/L)	25.05 ng/dL (SI: 0.87 nmol/L)			201.6-777.6 ng/dL (SI: 7-27 nmol/L)
E2-17 B	32 pg/mL, (SI: 117.44 pmol/L)			34 pg/mL, (SI: 124.78 pmol/L)			12.5-166 pg/mL, (SI: 45.87-609.22 pmol/L)
Cortisol	7.2 µg/dL (SI: 240 nmol/L)		2.94 µg/dL, (SI: 98 nmol/L)	6.27 µg/dL, (SI: 209 nmol/L)			5.13-16.08 µg/dL, (SI: 171-536 nmol/L)
DHEAS	65 µg/dL, (SI: 1.75 µmol/L)						98-340 µg/dL, (SI: 1.96-6.8 µmol/L)
ACTH	15 pg/mL, (SI: 3.3 pmol/L)						5-60 pg/mL, (SI: 1.1-13.2 pmol/L)
TSH	1.43 µIU/mL, (conventional and SI)						0.27-4.2 µIU/mL, (conventional and SI)
T4	10.75 µg/dL, (SI: 138.35 nmol/L)		4.7 µg/dL, (SI: 60.48 nmol/L)	5.1 µg/dL, (SI: 65.63 nmol/L)			4.8-12.7 µg/dL, (SI: 61.77-163.44 nmol/L)
T3	1.56 ng/mL, (SI: 0.02 nmol/L)						0.8-2.0 ng/mL, (SI: 0.012-0.03 nmol/L)
Imaging	4.2 × 3.6 × 2.9 cm	3.6 × 2.2 cm	3.4 × 6.8 × 2.85	4.5 × 3.4 × 3 cm	1.2 cm residue		
Comments	BRC 2.5 mg TDS	Sterotactic radiosurgery + BRC	CBG 0.5 mg twice a week	TNTS	CBG 0.25 mg once a week		

Abbreviations: BRC, bromocriptine; CBG, cabergoline; DHEAS, dehydroepiandrosterone sulfate; E2, estradiol; PRL, prolactin; SI, International System of Units; TDS, 3 times daily; TNTS, transnasal transsphenoidal surgery.

**Figure 1.**
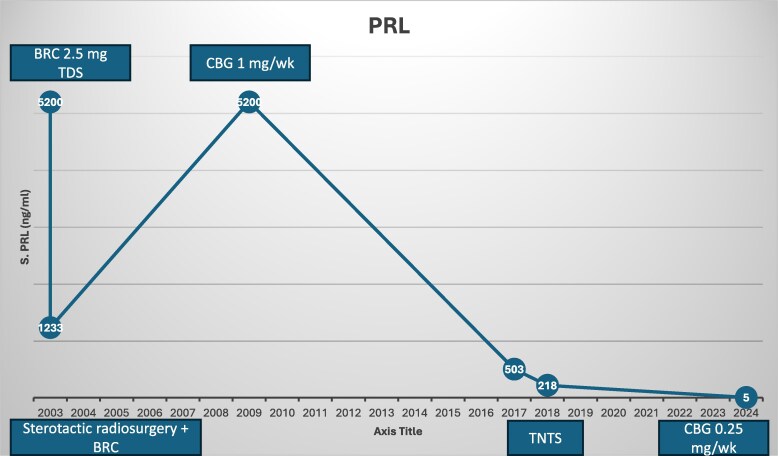
Serum PRL levels over time showing fluctuations in response to various treatment modalities. Abbreviations: BRC, bromocriptine; CBG, cabergoline; PRL, prolactin; TDS, 3 times daily; TNTS, transnasal transsphenoidal surgery.

The patient was started on BRC 2.5 mg 3 times a day. Three months later, his follow-up serum PRL had decreased to 1233 ng/mL (SI: 26136.6 mIU/L), with the trend of PRL (Fig. 1), while imaging revealed a reduction in tumor size to 3.6 × 2.2 cm. Around this time, in a multidisciplinary meeting after discussion with the patient, fractionated stereotactic radio-surgery (SRS) was performed (23 fractions 46 Gray) as he had refused surgery; the BRC dose was escalated to 5 mg 3 times daily in the interim period, which he continued regularly and regularly attended for follow-up every 6 months with the local general practitioner. He continued taking BRC with a prescription from a local practitioner until 2009. At that time represented at the age of 50 years with a serum PRL of 5200 ng/mL (SI: 110240 mIU/L) and a 3.4×6.8 × 2.85 cm sellar-suprasellar mass. Investigations revealed hypocortisolism and hypothyroidism (Table 1), and both he and his local doctor confirmed he had been fully compliant with his medication. His treatment was switched to cabergoline 0.5 mg twice weekly, along with the replacement of deficient hormones, in view of his poor response to his previous therapies.

**Figure 2.**
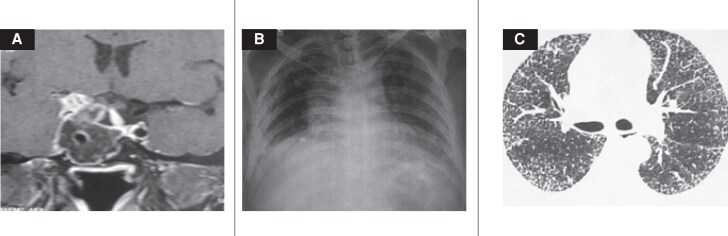
(A) Coronal T1-weighted MRI showing a sellar lesion with right parasellar extension consistent with a prolactin-secreting pituitary adenoma. (B) Chest radiograph demonstrating reticular opacities in the lower lung fields. (C) HRCT of the thorax revealing reticular thickening and honeycombing, suggestive of a usual interstitial pneumonia pattern. Abbreviations: HRCT, high-resolution computed tomography; MRI, magnetic resonance imaging.

**Figure 3.**
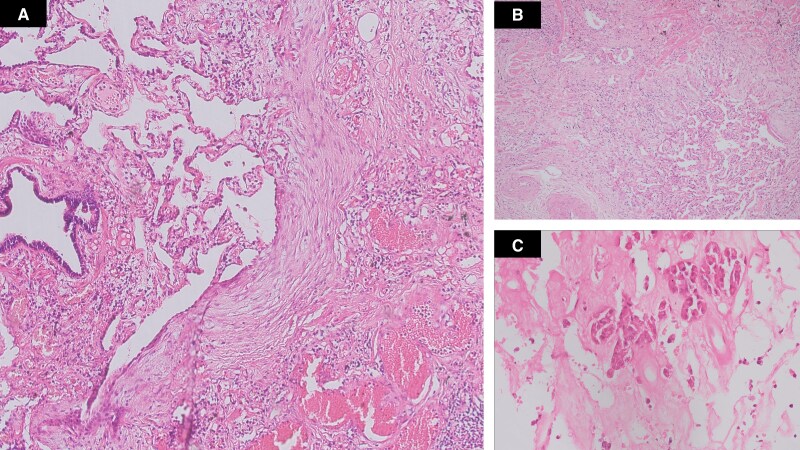
(A) High power showing hyalinized fibrosis with adjacent normal lung parenchyma and presence of fibroblastic foci at the junction of fibrosed and normal lung parenchyma (H&E, x200). (B) Low power showing fibrosis of lung parenchyma with architectural distortion (H&E, x20) (C) Section from pituitary adenoma, showing extensive fibrosis following treatment (H&E, x200). Abbreviation: H&E, hematoxylin and eosin.

The patient presented again to our hospital in 2017 at the age of 57 years. This time he was brought from prison due to indulgence in illegal gambling. A detailed history revealed he was also having many other impulse control disorders. During this period, he had continued cabergoline but was irregular with levothyroxine and hydrocortisone. He presented with headache, vomiting, and a recent onset of right-sided ptosis. Hormonal investigations are shown in Table 1; magnetic resonance imaging showed a 4.5 × 3.4 × 3.0 cm lesion with a predominantly right para-sellar extension, (Fig. 2A). Due to his impulse control disorder, the case was discussed in a multidisciplinary meeting, and the patient was briefed on the benefits and risks of available treatment options. He declined further medical therapy and now opted for surgical intervention. In the preoperative period, his PRL level was 503 ng/mL (SI: 10663.6 mIU/L). He underwent endoscopic transnasal transsphenoidal excision [6]. Histopathological examination revealed dense fibrosis and inflammatory infiltrates in the sellar tissue with no evidence of any apoplexy, as shown in Fig. 3C, and was negative for GH and showed focal positivity for PRL immunostaining with a Ki67 <1%. His IGF1 was 118 ng/mL (SI: 15.45 nmol/L) [reference range: 65.3-194 ng/mL (SI: 10.30-27.11 nmol/L)] and GH was 0.70 ng/mL (SI: 0.7 ug/L) [reference range: <2 ng/mL (SI: <2 ug/L)]. His immediate postoperative PRL was 2 ng/mL (SI: 42.4 mIU/L), which then increased to 217 ng/mL (SI: 4600.4 mIU/L) at 3 months. Postoperative imaging revealed a 1.2 cm right para-sellar residual lesion; SRS was not considered in view of its limited efficacy previously setting and patient refusal, and a modest dose of cabergoline 0.25 mg once-weekly was restarted (Fig. 2A).

In 2024, the patient presented with a 2-year history of progressive shortness of breath on exertion and a dry cough. He was a nonsmoker and reported no significant environmental exposure. The symptoms exacerbated during the winter months.

## Diagnostic Assessment

Cardiac evaluation, including electrocardiogram and 2-dimensional echocardiogram, was normal (ejection fraction 63%) without any valvular involvement. Plain chest radiology demonstrated reticular opacities in the lower lung fields (Fig. 2B). A high-resolution computed tomography of the thorax showed distorted lung architecture in both lungs and marked interstitial thickening, suggestive of early ILD (Fig. 2C). Pulmonary function tests indicated a restrictive pattern with a forced vital capacity (FVC) of 0.8 L (63% of predicted) and a forced expiratory volume 1/FVC ratio of 73%. A lung biopsy under radiological guidance revealed patchy areas of fibrosis in paraseptal and subbasal regions, with fibroblastic foci and eosinophilic collagenous material creating a honeycomb appearance (Fig. 3A and 3B).

Autoimmune markers including rheumatoid factor, anticyclic citrullinated peptide, antinuclear antibody, anti-Ro, La, Topo-isomerase, and small nuclear ribonucleic acid were largely negative except for myeloperoxidase antibody, which was weakly positive but on repeat with a quantitative method was negative, along with a normal serum angiotensin-converting enzyme level. The cumulative dose of bromocriptine received by the patient was approximately 19 100 mg, and the total cabergoline dose was approximately 490 mg. He was not on any other medication known to cause ILD. He was an accountant by profession with no significant clear environmental exposure, with a nonsmoking history, such that a drug-induced etiology was considered likely.

## Treatment

Cabergoline was discontinued, and the plan was to monitor serum PRL levels and imaging findings during prolonged follow-up. On the last follow-up, his PRL level is 0.79 ng/mL (SI: 16.74 mIU/L). His IGF-1 20.8 ng/mL (SI: 2.72 nmol/L). He is having all anti-pituitary hormone deficiencies and a repeat magnetic resonance imaging showing a very small right para-sellar residue.

## Outcome and Follow-up

Once he was diagnosed with UIP, he was started on a therapeutic dose of prednisolone 40 mg/day, which was gradually tapered down to 10 mg/day, and he has been off cabergoline for the last 12 months. He is on replacement with testosterone and levothyroxine. For his ILD, he is on the anti-fibrotic agent pirfenidone 200 mg 3 times daily, benzonatate for cough suppression, and prednisolone 10 mg/day, but without any oxygen therapy he has a normal arterial blood gas analysis.

## Discussion

We report a patient with a long-standing prolactinoma, diagnosed at 44 years, who received cumulative doses of BRC (19 100 mg) and cabergoline (490 mg) along with SRS and surgery. Despite tumor shrinkage and partial control of hyperprolactinemia, he eventually was diagnosed with ILD with a UIP pattern on biopsy. He had no significant environmental exposures, was a nonsmoker, and had negative autoimmune markers, thus raising the suspicion of drug-induced ILD.

Dopamine agonists are highly effective in the management of prolactinomas, Parkinson’s disease, Parkinson-related diseases, and even in acromegaly; however, ergot-derived agents exhibit activity at serotonin receptors triggering pathological fibrosis in the heart, lungs, pleura, and retroperitoneum [7]. Ergot-derived agents had higher reported odds ratios for fibrotic reactions, whereas non-ergot agents did not increase this risk. In the case of pleural/pulmonary fibrosis, the reported odds ratio was 12.3 (95% confidence interval, 7.1-19.7) with ergot-derived agents, while no influence was observed with non-ergot agents [8, 9]. The underlying mechanisms for predilection for a particular site of fibrosis are unknown.

Cabergoline and pergolide exhibit considerably higher receptor affinity compared with BRC and lisuride, thus correlating with a greater likelihood of fibrotic events [8-10]. This complication is most often documented in Parkinson’s disease cohorts, where higher daily and cumulative dose is used. In patients with prolactinomas, only a few isolated case reports have documented this adverse effect [11]. On the contrary, several prospective studies have not shown any such association [12]. In the histopathological examination of the pituitary tumor, there was extensive fibrosis, which could have been contributed by both SRS along with dopamine agonist therapy. Nevertheless, prolonged treatment, as seen in this case, may confer a risk of pulmonary fibrosis, as particularly other known predisposing factors were excluded. However, the cause-and-effect relationship cannot be established in our case as there are many unidentified factors. Cabergoline has been associated with pleural and pericardial fibrosis at doses of 1 to 10 mg per day for 4 months to 6 years [13], and pergolide has been linked to these complications at doses of 1 to 6 mg per day for 4 months to 11 years [14], while that with BRC has been found to be a dose exceeding 30 mg/day for 15 months to 10 years [15]. The non-ergot dopamine agonist quinagolide was not offered to the patient because of its nonavailability.

Hyperprolactinemia has also been shown to have fibrotic potential through a TGF-β-mediated pathway, which might have contributed to fibrosis in our case [16]. Although this patient had low levels of testosterone and dehydroepiandrosterone sulfate, this seems a less likely cause of fibrosis [17].

Although the clinical context strongly implicates dopamine agonist therapy as a potential cause of ILD in this patient, this is a coincidence. As the prescribed dose of the dopamine agonist was low, even though it was used for a long period of time, its role remains uncertain in our case [18]. A low dose of dopamine agonist used in the treatment of prolactinomas does not lead to fibrosis, and its direct role is controversial [19, 20]. During the preanesthetic checkup in 2017, the chest x-ray and pulmonary function of our patient were normal, but there was a gap of almost 7 years before the current presentation. The absence of alternative risk factors strengthens the likelihood of a drug-induced etiology; however, a causal relationship cannot be definitively established.

Timely recognition of these symptoms of ILD followed by chest imaging, pulmonary function tests may be indicated in selected cases [7]. On chest radiography, dopamine agonist-induced pulmonary fibrosis typically appears as reticular or reticulonodular opacities, predominantly in the lower lobes, while high-resolution computed tomography usually demonstrates patterns characteristic of UIP, such as peripheral and basal predominance, honeycombing, and reticular fibrotic changes. Pulmonary function tests typically show restrictive physiology (FVC <80% of predicted) and a reduced diffusion capacity for carbon monoxide. In certain instances, a lung biopsy may be necessary to confirm the pathological pattern of fibrosis (eg, fibroblastic foci, excessive collagen deposition) [12, 14].

Once ILD is suspected, prompt discontinuation or substantial reduction of the offending agent is essential, while also weighing the need for continued control of the primary disorder. Drug withdrawal can lead to clinical improvement in early cases. For patients with prolactinomas, alternative options, including pituitary surgery or radiotherapy, may be considered if the risks of ongoing dopamine agonist therapy are deemed unacceptable. Additionally, switching to a non-ergot derivative such as quinagolide may be contemplated. In the absence of other viable treatments or when withdrawal is not feasible, corticosteroid therapy may be an option for the treatment of interstitial lung disease. Otherwise, low-dose oxygen therapy or pulmonary rehabilitation may be considered [13]. Current guidelines for screening this rare complication are not well established [13].

Our case did not follow the standard protocol for managing macroprolactinoma due to the aggressive nature of the disease and the patient's decisions at various points.

In summary, this case underscores a rare yet significant complication of prolonged ergot-derived dopamine agonist therapy in a patient with prolactinoma. It highlights the need to consider drug-induced fibrosis in patients receiving long-term dopamine agonists, underscores the importance of close respiratory surveillance, and emphasizes prompt investigation of any new pulmonary symptoms. Further data from larger studies and registries are warranted to elucidate the dose-response relationship.

## Learning Points

- Long-term ergot-derived dopamine agonist therapy in prolactinoma may predispose to fibrotic complications, including ILD, despite generally lower dosing than in Parkinson’s disease.
- Early symptoms of ILD, such as exertional dyspnea and chronic cough, can be subtle. This underscores the importance of pulmonary function testing and imaging to identify progressive fibrosis only in symptomatic patients.
- Drug withdrawal or switching to alternative therapies (eg, non-ergot dopamine agonists, surgery, radiotherapy) may be warranted when drug-induced fibrotic changes are suspected.

## Contributors

R.S. contributed to the conception, clinical management, data acquisition, and drafting of the manuscript. A.B. provided histopathological analysis and interpretation of findings. D.A. contributed to the clinical assessment and review of the manuscript. J.G. contributed to clinical assessment of the patient and editing the manuscript. A.B.G. provided expert insights into the endocrinological aspects and critically revised the manuscript for important intellectual content. P.D. supervised the patient management, ensured the integrity of the work, and contributed to the final manuscript’s preparation and approval. All authors reviewed and approved the final manuscript for submission.

## Data Availability

Data sharing is not applicable to this article as no datasets were generated or analyzed during the current study.
